# Genetic Engineering for Cereal Crop Yield Improvement and Disease Resistant Breeding

**DOI:** 10.1155/tswj/6743917

**Published:** 2025-03-05

**Authors:** Asaye Demelash Limenie, Mastewal Alehegn

**Affiliations:** College of Agriculture and Natural Resource, Debre Markos University, Debre Markos, Ethiopia

**Keywords:** crop, disease, genetic engineering, resistant breeding, yield

## Abstract

Genetic engineering has revolutionized the field of agriculture, providing innovative solutions to enhance crop productivity and resilience against diseases. Among the various crops, cereals hold a pivotal position in global food security, feeding a significant portion of the world population. Genetic engineering, in cereal crop breeding, has opened new avenues for yield improvement and the development of disease-resistant varieties. Growing population and climate change, traditional breeding methods alone are insufficient to meet the increasing demand for food while ensuring sustainability. Genetic engineering offers a precise and efficient approach to introduce desirable traits into cereal crops, thereby improving yield and reducing the impact of diseases. One of the primary objectives of genetic engineering in cereal crops is to enhance yield potential. This can be achieved by modifying genes associated with key traits such as photosynthetic efficiency, stress tolerance, and nutrient use efficiency. For instance, genetic engineering can be used to increase the efficiency of light capture and conversion into biomass, thereby boosting yield. Disease resistance is another critical area where genetic engineering can make a significant impact. Pathogens and pests pose a constant threat to cereal crops, leading to significant yield losses. Genetic engineering techniques allow the introduction of genes encoding resistance to diseases, such as those from wild relatives or from other organisms. Here, evidence shows that the incorporation of *Bacillus thuringiensis* (*Bt*) genes into maize has effectively controlled corn borer infestations, reducing the need for chemical pesticides. This not only reduces yield losses but also minimizes the development of pathogen resistance to single-gene interventions.

## 1. Introduction

Cereals are grasses that are grown for their edible parts and belong to Poaceae, commonly referred to as Gramineae. The principal crops used to make staple cereals are barley, oats, wheat, rice, maize, sorghum, and millet [1]. The endosperm, germ, and bran of these cereals are the edible parts of their caryopsis that are grown [2]. These plants have adapted to survive in conditions where they are often subjected to a variety of stressors, including dryness, high temperatures, salt, mineral toxicity, and a lack of water. Intended for maximizing cereal crop yield and breeding activities, genetic engineering is an influential implement in the field of agriculture, offering significant potential for improving disease resistance [3]. Cereals are vital components of the global food system, providing a significant portion of the world's dietary calories and protein. With the growing population and the challenges posed by climate change, genetic engineering offers innovative solutions to enhance crop productivity and resilience against diseases [4]. The objective of genetic engineering in cereal crops is to improve yield potential and develop disease-resistant varieties [5]. This can be achieved by modifying genes associated with key traits such as photosynthetic efficiency, stress tolerance, and nutrient use efficiency. Genetic engineering techniques allow the introduction of genes encoding resistance to diseases, such as those from wild relatives or from other organisms [6]. It has an ability to stack multiple disease-resistant genes into a single cereal variety, providing broad-spectrum resistance [7], not only reducing yield losses but also minimizing the development of pathogen resistance to single-gene interventions. However, the application of genetic engineering in cereal crop breeding is not without challenges. Regulatory frameworks vary across countries, and public perception can influence the adoption of genetically modified (GM) crops [8], the environmental impact of GM crops, and the potential for gene flow to wild relatives. Despite these challenges, genetic engineering represents a promising strategy for improving cereal crop yield and breeding for disease resistance [9]. As research continues to advance, the integration of genetic engineering into cereal crop breeding programs is likely to play an increasingly important role in meeting the food demands of the future [10].

### 1.1. Overview of the Global Importance of Cereal Crops

Cereal crops are among the most important agricultural commodities globally, playing a pivotal role in global food security and human nutrition [11], the primary sources of dietary calories and protein for a significant portion of the world's population, particularly in developing countries. Wheat, for instance, is a staple food for over a third of the world's population, providing approximately 20% of the calories and protein consumed by humans [12]. Rice is another critical cereal, particularly in Asia and parts of Africa, where it is a primary source of nutrition for over half of the world's population [11]. Maize, while less of a staple in human diets, is a crucial feed for livestock and a key ingredient in many processed foods. Barley, while not as widely consumed as the others, is also an important food source and is used in animal feed and the brewing industry [13]. The global production and consumption of cereal crops are significant indicators of economic development and food security [14]. These crops are grown on a massive scale, with global production of wheat, rice, and maize alone exceeding 600 million metric tons annually. The demand for cereal crops is expected to increase significantly in the coming decades due to population growth, urbanization, and changing dietary patterns [15]. Moreover, cereal crops are essential for food aid and emergency relief operations, making them crucial for global food security and stability. The ability to produce high-yielding, disease-resistant cereal varieties through genetic engineering can significantly impact global food security by increasing crop productivity and resilience against biotic and abiotic stresses [3, 16, 17].

### 1.2. Challenges Faced by Cereal Crop Production: Biotic and Abiotic Stressors

As Table 1 indicates, there are several obstacles in the way of producing cereal crops, such as biotic and abiotic stress, which may have a big influence on yield and quality [18, 19]. Abiotic stressors, which are nonliving environmental variables like drought, salt, temperature extremes, and nutrient shortages, and biotic stressors, which are caused by live creatures like pests, diseases, and weeds, are the two basic groups into which these stressors may be divided [20]; insects, nematodes, and mites can cause significant damage to crops, reducing yield and quality. Pathogens, such as fungi, bacteria, and viruses, can also infect cereal crops, leading to diseases that can devastate entire crops [21]. Weeds compete with cereal crops for resources such as water, nutrients, and light, reducing crop productivity [22]. Drought is a major concern, as cereal crops are sensitive to water stress, which can lead to reduced growth, yield, and quality [20]. Salinity is another significant issue, particularly in arid and semiarid regions, where high soil salinity can limit crop growth and productivity. Temperature extremes, including heat stress and cold stress, can also affect cereal crop growth and development, leading to reduced yield and quality [23]; nutrient deficiencies, such as deficiencies in nitrogen, phosphorus, and potassium, can also limit crop productivity [24]. Genetic engineering offers a promising strategy for enhancing cereal crop resilience against these stressors. Using genetic engineering, agricultural yield and sustainability may be enhanced by adding genes that give resistance to pests, diseases, and abiotic challenges. To reduce the need for chemical pesticides and fertilizers, genetic engineering, for instance, may be used to create crops that are resistant to diseases and pests. Genetic engineering can also be used to develop crops that are more tolerant to abiotic stresses such as drought and salinity, improving crop productivity in marginal environments [25, 26].

### 1.3. The Potential of Genetic Engineering in Addressing Challenges

Genetic engineering offers significant potential for addressing the challenges faced by cereal crop production, including biotic and abiotic stressors (Table 2). By manipulating the genetic makeup of cereal crops, scientists can develop new varieties that are more resistant to pests, diseases, and environmental stresses, leading to higher yields and improved food security [27, 28], in which genetic engineering can address these challenges is by introducing genes that confer resistance to pests and diseases. For example, *Bt* corn, which is genetically engineered to produce a protein that is toxic to certain insects, has been widely adopted by farmers and has significantly reduced the use of insecticides [29]. Similarly, genetically engineered crops that are resistant to diseases caused by fungi, bacteria, and viruses can reduce the need for chemical pesticides [30], can be expensive and environmentally damaging, and pose risks to human health. Genetic engineering can also be used to develop crops that are more tolerant to abiotic stresses such as drought, salinity, and temperature extremes [26]. Genetically engineered crops that are more efficient in their use of water can help to mitigate the impacts of drought, while crops that are more tolerant to salinity can be grown in soils that are unsuitable for conventional agriculture. In addition to improving crop resilience, genetic engineering can also be used to enhance the nutritional content of cereal crops [31]. For example, golden rice, which is genetically engineered to produce beta-carotene, a precursor to vitamin A, has the potential to reduce vitamin A deficiency, a major public health problem in many developing countries. However, the potential of genetic engineering in addressing the challenges faced by cereal crop production is not without controversy. There are concerns about the environmental impact of genetically engineered crops, including the potential for gene flow to wild relatives and the development of herbicide-resistant weeds [32]. There are also concerns about the safety of genetically engineered foods, although many scientific studies have concluded that they are safe for human consumption. Despite these challenges, genetic engineering offers significant potential for improving cereal crop production and addressing the challenges of global food security [33]. As such, it is likely to play an increasingly important role in the development of new varieties of cereal crops that are more resilient, productive, and nutritious [34].

## 2. Historical Context of Cereal Crop Improvement

The history of cereal crop improvement is a long and complex one, dating back to the early days of agriculture. Over the centuries, farmers and plant breeders have developed new varieties of cereal crops that are more productive, resilient, and adapted to different growing conditions [35]. The earliest forms of cereal crop improvement were likely through traditional breeding methods, such as selecting plants with desirable traits and cross-breeding them to produce new varieties. For example, early farmers in the Fertile Crescent may have selected wheat plants with larger seeds and better disease resistance and crossbred them to produce new varieties that were more productive and adapted to local growing conditions. In the 20th century, the development of new plant breeding techniques, such as hybridization and mutation breeding, accelerated the pace of cereal crop improvement [36]. Hybridization involves crossing two different varieties of a crop to produce offspring with improved traits, while mutation breeding involves exposing plants to radiation or chemicals to induce mutations that can lead to new and improved traits. The Green Revolution of the 1950s and 1960s was a major turning point in the history of cereal crop improvement [37]. This period saw the development of new, high-yielding varieties of cereal crops that were more responsive to fertilizers and pesticides and could be grown in a wider range of environments. These new varieties played a key role in increasing global food production and reducing hunger and malnutrition in many parts of the world [38]. The advent of genetic engineering in the latter half of the 20th century opened up new possibilities for cereal crop improvement. Genetic engineering allows scientists to introduce specific genes into cereal crops, conferring traits such as pest and disease resistance, herbicide tolerance, and improved nutritional content [30]. Despite these advances, cereal crop improvement remains a challenging and ongoing process. The increasing prevalence of pests, diseases, and environmental stresses, as well as the need to produce more food with fewer resources, requires continued innovation and investment in crop improvement research [3, 39].

### 2.1. Traditional Breeding Methods and Their Limitations

Traditional breeding methods have been used for centuries to improve crop varieties, including the development of disease-resistant varieties. These methods involve crossing plants with desirable traits, such as resistance to a particular disease, and selecting the offspring that inherit these traits [35]. However, traditional breeding has its limitations, particularly when it comes to developing disease-resistant crops. One of the main limitations of traditional breeding is that it can be time-consuming and labor-intensive ([40]; it can take many years to develop a new crop variety through traditional breeding, as multiple generations of plants may need to be grown and evaluated before a plant with the desired traits is identified. This can be also a significant barrier to the rapid development of disease-resistant crops, especially in the face of emerging diseases that threaten crop production. Another limitation of traditional breeding is that it can be challenging to introduce genes for disease resistance from one species into another [41]. Many of the genes that confer disease resistance are species-specific, meaning that they are not easily transferable between different species of plants [42], and can limit the ability of breeders to develop disease-resistant crops using traditional breeding methods. In addition, traditional breeding can sometimes result in unintended consequences, such as the introduction of undesirable traits along with the desired ones. For example, a plant that is resistant to a particular disease may also have reduced yield or other negative traits that make it less desirable for farmers. Despite these limitations, traditional breeding methods remain an important tool in the development of disease-resistant crops. By carefully selecting and crossing plants with desirable traits, breeders can develop new varieties that are better able to withstand disease pressures [43]. In recent years, advances in genetic engineering have opened up new possibilities for the development of disease-resistant crops. Genetic engineering allows scientists to introduce specific genes into crops, conferring resistance to diseases without the limitations of traditional breeding. According to Islam et al. [44], genetically engineered crops can be developed to express proteins that are toxic to specific pests or pathogens or to have enhanced disease-fighting mechanisms. Traditional breeding methods have significant role in the development of disease-resistant crops, but they have their limitations. The development of new and improved varieties of crops will require a combination of traditional breeding methods and modern genetic engineering techniques, as well as continued investment in research and development [45]. In general, leveraging the strengths of approaches, scientists and plant breeders can continue to develop crops that are better able to withstand disease pressures and meet the food needs of a growing global population [46].

### 2.2. The Advent of Molecular Biology and Genetic Engineering

The advent of molecular biology and genetic engineering has revolutionized the field of plant breeding, particularly in the development of disease-resistant crops. These technologies have provided breeders with new tools and approaches for introducing disease resistance into crops, overcoming many of the limitations of traditional breeding methods [45]. One of the key advances in molecular biology and genetic engineering for disease-resistant breeding has been the identification and characterization of genes that confer resistance to specific diseases. Using molecular techniques such as DNA sequencing and gene cloning, scientists can identify genes that are responsible for disease resistance in one species and introduce them into another species through genetic engineering [47]. Genetic engineering allows for the precise introduction of these disease-resistant genes into crops, without the need for time-consuming and labor-intensive traditional breeding methods [48], and can result in the rapid development of new crop varieties with improved disease resistance, which can be particularly important in the face of emerging diseases that threaten crop production. In addition to the introduction of disease-resistant genes, genetic engineering can also be used to enhance the natural disease-fighting mechanisms of plants [49]. For example, genetically engineered crops can be developed to produce higher levels of antimicrobial compounds or to have improved immune systems that are better able to recognize and defend against pathogens. Molecular biology and genetic engineering have also made it possible to develop crops with multiple disease-resistant traits, which can be particularly important for managing complex diseases that are caused by multiple pathogens [50]. By combining genes for resistance to different diseases, breeders can develop crops that are more broadly resistant to a range of pathogens. Despite these advances, the use of molecular biology and genetic engineering in disease-resistant breeding is not without its challenges and limitations. There are concerns about the environmental impact of genetically engineered crops, including the potential for gene flow to wild relatives and the development of herbicide-resistant weeds [51]. There are also concerns about the safety of genetically engineered foods, although many scientific studies have concluded that they are safe for human consumption.

### 2.3. Milestones in Genetic Engineering of Cereal Crops

The genetic engineering of cereal crops has made significant advances since the early days of the technology. Introduction of *Bacillus thuringiensis* (*Bt*) maize (1996) of *Bt* genes were first introduced into maize to confer resistance against the European corn borer [52]. This was a significant milestone as it marked the first commercialization of a genetically engineered cereal crop. Development of herbicide-tolerant crops (1996) [53]: the introduction of crops resistant to glyphosate, such as soybeans and corn, revolutionized weed management in agriculture. This technology was later adapted for cereal crops, including corn and wheat, allowing for more efficient and environmentally friendly weed control. Stacked traits in maize (2003): The development of maize varieties with multiple, or “stacked,” traits, such as resistance to both insects and herbicides, marked a significant advancement in genetic engineering [54]. This allowed for more comprehensive management of pests and weeds in cereal crops. Development of drought-tolerant maize (2013): The introduction of genetically engineered maize varieties that are more tolerant to drought conditions represents a major step forward in addressing one of the most pressing challenges in agriculture [10]. Genome editing in cereal crops: The advent of genome editing technologies, such as CRISPR-Cas9, has opened up new possibilities for genetic engineering in cereal crops [55]. These technologies allow for precise modifications to the genome, making it possible to introduce targeted changes without the need for traditional transgenic approaches. Development of disease-resistant wheat (ongoing): Efforts are underway to develop genetically engineered wheat varieties that are resistant to diseases such as rust. This could help to increase yields and reduce the need for fungicides in wheat production. Nutritionally enhanced cereal crops: Researchers are working on developing cereal crops with improved nutritional content, such as higher levels of protein, vitamins, or minerals. Genome-wide association studies (GWAS) in cereal crops (2000s): The application of GWAS has allowed for the identification of genetic markers associated with important traits in cereal crops [56]. This information can be used to guide breeding efforts and accelerate the development of new varieties through marker-assisted selection. Development of nontransgenic gene editing (2010s): Advances in gene editing techniques have made it possible to make precise changes to the genome without the introduction of foreign genes. This has helped to address some of the public concerns associated with transgenic crops. Genome sequencing of cereal crops (2000s): The completion of the genome sequences for major cereal crops, such as maize, rice, and wheat, has provided a wealth of information for breeders and geneticists. This has facilitated the identification of genes associated with important traits and the development of new breeding strategies [57]. The milestones highlight the rapid pace of technological advancement in the genetic engineering of cereal crops. As these technologies continue to evolve, they are likely to play an increasingly important role in addressing the challenges facing global agriculture, including climate change, resource limitations, and food security.

## 3. Genetic Engineering for Yield Improvement

Genetic engineering has emerged as a powerful tool for improving crop yield, a critical aspect of global food security. The primary goal of genetic engineering in this context is to enhance the productivity of cereal crops, such as rice, wheat, and maize, under various environmental conditions. This review is aimed at summarizing the current state of genetic engineering for yield improvement, highlighting the strategies, achievements, and challenges. Genetic engineering has enabled the introduction of genes encoding for proteins that confer resistance to pests and diseases [50]. For instance, *Bt* genes have been introduced into maize and cotton to protect against lepidopteran pests [58]. Abiotic stresses, such as drought, salinity, and temperature extremes, significantly affect crop yield. Genes that enhance stress tolerance, such as those encoding for aquaporins, late embryogenesis abundant (LEA) proteins, and transcription factors, have been introduced into cereal crops [59–61]. Improving nutrient use efficiency can reduce the need for fertilizers and enhance yield. Genes involved in nitrogen, phosphorus, and water use efficiency have been targeted for genetic engineering. Modifying photosynthetic pathways to increase efficiency can lead to higher yields. Genes encoding for enzymes involved in photosynthesis, such as Rubisco, have been engineered to improve their activity and stability [62]. Genes encoding for enzymes that confer tolerance to herbicides have been introduced into crops, allowing for more effective weed control [63]. Genetic engineering can also improve the nutritional quality and shelf life of cereal crops, making them more valuable and potentially increasing yield through higher market demand. Genetic engineering has led to the development of high-yielding varieties of cereal crops. For example, *Bt* cotton has significantly reduced the use of insecticides and increased yield in many countries. Similarly, GM crops with improved stress tolerance have shown promise in field trials, demonstrating increased yield under adverse conditions [64]. Despite the achievements, several challenges remain. One of the major challenges is the regulatory framework, which varies significantly among countries and can slow down the adoption of genetically engineered crops. Public perception and acceptance of GM crops are also significant hurdles, with concerns about the environmental impact and safety for human consumption [65].

## 4. Disease Resistance Breeding Through Genetic Engineering

Disease-resistant breeding through genetic engineering has great use for plant breeders to combat pathogens that cause significant losses in crop yield and quality worldwide [50]. This approach involves the intentional modification of an organism's genetic material to enhance its ability to resist diseases caused by various pathogens [66]. Genetic engineering allows for the precise introduction of disease-resistant genes into a plant's genome, bypassing the lengthy and often unpredictable process of traditional breeding. This can significantly accelerate the development of disease-resistant crops. Genetic engineering can introduce resistance to multiple diseases simultaneously, which is not always feasible with traditional breeding methods. This is particularly useful in combating complex diseases that are caused by multiple pathogens. Genetic engineering allows for the transfer of genes from distantly related species, which is not possible or very difficult with traditional breeding methods [67]. This opens up the possibility of introducing novel disease-resistant traits from a wide range of sources. By reducing the need for chemical pesticides, genetically engineered crops with disease resistance can lead to a more sustainable agriculture, benefiting both the environment and human health [68]. Disease resistance breeding through genetic engineering offers significant potential for improving crop resilience against pathogens. However, its adoption and impact are contingent upon addressing the challenges and concerns associated with this technology. As with any new technology, a balanced approach that considers the environmental, social, and economic implications is crucial for its responsible and beneficial use in agriculture [69, 70].

### 4.1. Mechanisms of Disease Resistance in Plants

Plants have evolved various mechanisms to defend themselves against pathogens. Innate immunity is the first line of defense that plants use to recognize and respond to pathogens [71], a nonspecific defense mechanism that is present in all plants and does not require prior exposure to the pathogen [72]. The plant cell wall acts as a physical barrier against pathogens. It is composed of cellulose, hemicellulose, and pectin, which are difficult for pathogens to degrade. Additionally, plants produce secondary metabolites such as lignin, suberin, and callose, which strengthen the cell wall and make it more resistant to pathogen attack. Pathogen-associated molecular patterns (PAMPs) are conserved molecules found in many pathogens, while pattern recognition receptors (PRRs) are receptors that are specific [73]. The recognition of PAMPs by PRRs leads to a PAMP-triggered immunity (PTI) response, which includes the production of reactive oxygen species (ROS), callose deposition, and the activation of defense-related genes [74, 75].

#### 4.1.1. Acquired Resistance

Acquired resistance is a specific defense mechanism that is induced by the plant after it has been exposed to a pathogen. It is a more robust form of defense than innate immunity and can provide long-lasting protection against a wide range of pathogens. Acquired resistance can be divided into two main types: systemic acquired resistance (SAR): This is a broad-spectrum resistance that is induced by the plant in response to a pathogen attack. It is characterized by the systemic production of salicylic acid (SA), which leads to the expression of defense genes and the accumulation of pathogen-related (PR) proteins [76]. SAR provides long-lasting protection against a wide range of pathogens. RNA-mediated silencing (RMS): This is a form of posttranscriptional gene silencing that is triggered by the presence of double-stranded RNA (dsRNA). Pathogens can produce dsRNA, which can be recognized by the plant and used to silence the expression of genes that are essential for pathogen survival. RMS is a highly specific form of defense that can provide long-lasting protection against a wide range of pathogens [77].

#### 4.1.2. Genetic Engineering for Disease Resistance

Genetic engineering can be used to enhance the natural defense mechanisms in plants. Genes encoding PRRs, PR proteins, and other defense-related proteins can be introduced into plants to enhance their innate immunity [78, 79]. Genes encoding proteins that are involved in SAR and RMS can also be introduced into plants to enhance their acquired resistance [80]. In addition, genetic engineering can be used to introduce novel disease-resistant genes into plants [50]. Genes encoding proteins that are toxic to pathogens can be introduced into plants. These proteins can kill or inhibit the growth of pathogens, providing the plant with a high level of protection against disease [81]. In understanding the mechanisms of disease resistance in plants and how they can be enhanced through genetic engineering, it is possible to develop new strategies for improving the health and productivity of crops [3, 50].

### 4.2. Intellectual Property Rights (IPRs) and Their Impact on Breeding Programs

IPRs play a significant role in breeding programs, particularly in the agricultural sector, where they can affect the development, distribution, and adoption of new plant varieties [82]. IPRs, such as patents and plant variety protection (PVP) rights, provide breeders with exclusive rights to their creations for a certain period. This exclusivity can serve as an incentive for private companies and public institutions to invest in breeding research, as they can recoup their investment through the sale of seeds or licensing agreements. IPRs protect the intellectual efforts and resources invested in developing new plant varieties [83]. This protection can deter others from copying the variety or its traits without permission, ensuring that the breeder can benefit from their work. Breeders can use IPRs to control the distribution and sale of their varieties, which can be crucial for commercial success. This control allows breeders to set prices, negotiate licensing agreements, and manage the release of their varieties into the market [84], which can also impact the accessibility of new varieties. For example, patents on genetically modified organisms (GMOs) or certain traits can limit the ability of small-scale farmers or public breeding programs to access these innovations, potentially leading to concerns about biopiracy or the monopolization of seed markets. The presence of IPRs can affect the willingness of breeders to share germplasm and breeding materials. Some institutions or breeders may be reluctant to share materials if they fear losing control over the intellectual property of any resulting varieties [85]. The impact of IPRs can be different for public and private breeding programs. Public institutions may focus on breeding for public good, such as developing varieties resilient to climate change or disease, and may prioritize open access to their materials. In contrast, private companies often prioritize varieties with commercial value and may rely more heavily on IPRs to protect their investments. IPRs can influence the regulatory environment for breeding. For instance, the complexity of IPRs and the potential for litigation can increase the cost and time required for regulatory approval of new varieties, which can impact the pace of innovation. The application of IPRs to new breeding techniques, such as gene editing, is a current area of debate [86]. The extent to which gene-edited crops should be regulated and whether they should be patentable is a topic of discussion among policymakers, scientists, and the public. Lately, IPRs are a complex issue in breeding programs, influencing the investment in breeding research, the accessibility of new varieties, and the collaboration among breeders [87]. Striking the right balance between protecting the interests of breeders and ensuring the availability of new varieties for the public good is a key challenge in the field of plant breeding.

### 4.3. Biosafety Protocols and Risk Assessment

Biosafety and regulatory considerations are crucial aspects of genetic engineering in cereals, ensuring that the technology is used in a responsible and sustainable manner [28], and the primary goal is to protect human health and the environment while facilitating innovation in agriculture [88]. An international treaty that is aimed at ensuring the safe handling, transport, and use of living modified organisms (LMOs), resulting from modern biotechnology [89], requires countries to establish biosafety frameworks and to regulate the transboundary movement, import, and release of LMOs into the environment [90]. A mechanism is established by the Cartagena Protocol to facilitate the exchange of information on LMOs. Each country has its own national Biosafety Clearing House (BCH) to provide information on regulations, decisions, and public awareness activities related to GMOs. Many countries have established regulatory bodies to oversee the development, testing, and release of GM crops [91]. These bodies typically include a combination of government agencies responsible for biosafety, environment, health, and agriculture. Before a GM crop can be released into the environment, it must undergo a rigorous risk assessment to evaluate its potential impact on human health and the environment. This includes assessments of potential adverse effects on biodiversity, potential for gene flow to wild relatives, and impacts on nontarget organisms. Measures are in place to prevent the unintended release of GM crops during research and development. Containment facilities are designed to prevent the escape of GMOs into the environment [92, 93]. Many countries have regulations in place for the labeling of GM foods. Labeling requirements vary, but they generally aim to provide consumers with information about the presence of GM ingredients in food products. Postrelease monitoring is conducted to track the performance of GM crops in the field and to detect any unintended effects on the environment or human health [94].

Biosafety protocols and risk assessment are crucial components of modern genetic engineering. These protocols are designed to ensure the safe handling, manipulation, and use of organisms, genetic material, and associated technologies in a way that minimizes the potential risks to humans, the environment, and the economy. Biosafety protocols are sets of guidelines and procedures that provide a framework for the safe conduct of research and development activities involving living organisms, including GMOs [95]. These protocols are typically developed and implemented by research institutions, industry, and regulatory bodies. They cover various aspects of biosafety. Laboratory safety includes the proper handling and disposal of biological materials, the use of personal protective equipment (PPE), and the containment of potentially hazardous organisms [96]. Transportation safety includes procedures for the safe transport of biological materials to prevent contamination or exposure during transit. Environmental safety: measures prevent the unintentional release of GMOs into the environment and to assess the potential impact of such releases. Public health safety: assessments of the potential risks to human health from exposure to GMOs or their products. Risk assessment is a systematic process for evaluating the potential risks associated with a particular activity or technology. Hazard identification: identifying the potential hazards associated with a particular organism or technology, such as the potential for it to cause harm to humans, animals, plants, or the environment. Exposure assessment: determining the likelihood of exposure to the hazard, including the routes and levels of exposure. Risk characterization: evaluating the severity of the potential harm and combining this with the likelihood of exposure to determine the overall risk. Risk management: developing and implementing strategies to prevent, control, or mitigate the risks identified in the assessment. National and international regulatory frameworks play a key role in ensuring biosafety. These frameworks establish guidelines and regulations for the development, testing, and commercialization of GMOs [97]. They also provide for the oversight and enforcement of biosafety protocols and the conduct of risk assessments. Several international agreements and conventions address biosafety and risk assessment, including the Cartagena Protocol on Biosafety, which provides a framework for the safe handling, transport, and use of LMOs resulting from modern biotechnology [92]. Public engagement is an important aspect of biosafety and risk assessment. Stakeholders, including the public, should have opportunities to participate in the development and implementation of biosafety protocols and the conduct of risk assessments. This can help to ensure that these processes are transparent, inclusive, and responsive to public concerns. Biosafety protocols and risk assessment are essential for ensuring the safe and responsible use of biotechnology and genetic engineering [98]. They help to protect human health, the environment, and the economy, while also supporting the development of beneficial products and technologies.

## 5. Future Perspectives and Emerging Technologies

Genetic engineering has revolutionized the field of agriculture, offering unprecedented opportunities for improving cereal crop yield and enhancing disease resistance. As we look towards the future, several emerging technologies and perspectives are likely to shape the landscape of genetic engineering for cereal crop improvement. Technologies like CRISPR-Cas9 have made it possible to make precise, targeted edits to the genome [99]. This allows for the introduction of disease-resistant genes, the removal of undesirable traits, and the enhancement of yield-related characteristics with high precision. By analyzing the genetic variations across a large number of individuals, GWAS can identify genes and genetic markers associated with important traits. This information can be used to breed crops with improved yield and disease resistance. This field involves designing and engineering biological systems that do not exist in nature. In agriculture, synthetic biology can be used to create crops with novel traits, such as enhanced nutrient content, improved stress tolerance, and increased yield. The integration of genetic engineering with precision agriculture technologies can lead to more efficient use of resources and improved crop management. For example, genetically engineered crops could be designed to respond to specific environmental conditions, reducing the need for manual intervention [9, 30]. Understanding how environmental factors can influence gene expression without changing the DNA sequence itself opens up new avenues for crop improvement. By manipulating epigenetic marks, it may be possible to enhance stress tolerance and disease resistance. Plant microbiome plays a crucial role in plant health and productivity. Genetic engineering could be used to introduce beneficial microbes into cereal crops, enhancing their ability to withstand disease and environmental stress [100]. As climate change poses new challenges to agriculture, genetic engineering can be used to develop crops that are more resilient to drought, heat, and other climate stresses. This approach uses genetic markers to predict the performance of individual plants, allowing breeders to select the best candidates for breeding without waiting for the plants to mature, and can accelerate the breeding process and improve the efficiency of genetic engineering efforts [4, 101]. Advances in computational biology and machine learning are enabling more accurate predictions of how genetic changes will affect plant traits. This can guide the design of genetic engineering experiments and improve the efficiency of crop improvement programs. As genetic engineering technologies advance, there will be a need for updated regulatory frameworks and public education to ensure that these technologies are adopted in a responsible and sustainable manner [100].

## 6. Future Directions for Research and Development in Cereal Crop Genetic Engineering

Advances in genome editing technologies, such as CRISPR-Cas9, will continue to drive innovation in cereal crop improvement. Research will focus on developing more precise and efficient editing tools, as well as on understanding the off-target effects of technologies. The integration of synthetic biology with cereal crop genetics could lead to the development of novel traits, such as enhanced nutrient content, improved stress tolerance, and increased yield. Research will focus on designing and testing synthetic biological systems in cereal crops. The plant microbiome plays a crucial role in plant health and productivity. Research will focus on identifying beneficial microbes and developing strategies for their introduction into cereal crops. As climate change poses new challenges to agriculture, research will focus on developing cereal crops that are more resilient to drought, heat, and other climate stresses. Advances in computational biology and machine learning will continue to drive innovation in genetic engineering. Research will focus on developing more accurate predictive models and tools for genetic engineering. There is a growing interest in developing cereal crops with improved nutritional quality. Research will focus on identifying genes and genetic pathways that can enhance the nutritional content of cereal crops. There will be a continued emphasis on developing genetic engineering strategies that support sustainable agriculture practices, including reduced reliance on synthetic inputs and maintenance of biodiversity. These future directions for research and development in cereal crop genetic engineering will be critical for addressing the challenges of global food security, climate change, and sustainable agriculture. However, they will also require careful consideration of the ethical, environmental, and social implications of these technologies.

## 7. Conclusion

Genetic engineering has the potential to significantly improve cereal crop yield and enhance disease resistance, but its success depends on addressing the challenges associated with regulation, public perception, and IPRs. Collaboration among different institutions, policymakers, industry, and the public is crucial to ensure that genetic engineering is used responsibly and effectively to enhance global food security at large.

## Figures and Tables

**Table 1:** Impact of biotic and abiotic stressors.

**Type of stressor**	**Specific challenge**	**Impact on crop production**
Biotic stressors	Pests and diseases	Reduced yield and crop quality
	Weeds	Competition for nutrients, water, and light
	Fungal infections	Damage to roots, stems, and leaves
	Insect infestations	Direct feeding and disease vectoring

Abiotic stressors	Drought	Water scarcity affecting growth
	Flooding	Root oxygen deficiency and rot
	Soil degradation	Loss of fertility and structure
	Temperature extremes (heat/cold)	Disruption in growth and development
	Nutrient deficiency	Inadequate nutrients for healthy growth
	Salinity	Reduced water uptake and toxicity

**Table 2:** Challenges cereal crops face and the role of genetic engineering.

**Challenge**	**Genetic engineering approach**	**Expected outcomes**
Drought stress	Introduction of drought-tolerance genes	Enhanced water-use efficiency and crop yield under low-water conditions
Salinity stress	Expression of salt-tolerance genes	Improved growth and productivity in saline soils
Pests and Diseases	Development of pest-resistant crops using *Bt* genes or RNA interference	Reduced pesticide use, lower crop losses, and sustainable pest management
Nutrient deficiency	Biofortification with genes for nutrient synthesis (e.g., Golden Rice)	Enhanced nutritional content, addressing malnutrition
Low yield potential	Manipulation of genes controlling photosynthesis	Increased photosynthetic efficiency and higher biomass production
Herbicide tolerance	Engineering crops with herbicide-resistance genes	Better weed management, reduced competition for resources, and higher productivity
Disease resistance	CRISPR/Cas9-edited resistance genes for pathogens	Targeted resistance against specific fungal, bacterial, or viral infections
Postharvest losses	Modifying genes affecting ripening and spoilage (e.g., polygalacturonase inhibitors)	Extended shelf life and reduced losses during storage and transportation
Soil nutrient management	Nitrogen-use efficiency genes	Reduced dependency on chemical fertilizers and improved sustainability of farming practices

## Data Availability

It is a review article, and we will not expect newly generated data from such manuscript because we used secondary data.

## References

[1] Gizaw W., Assegid D. (2021). Trend of cereal crops production area and productivity, in Ethiopia. *Journal of Cereals and Oilseeds*.

[2] das Graças Costa E., de Souza P. M. (2023). Introduction to cereals. *Cereal-Based Food Products*.

[3] Bigini V., Camerlengo F., Botticella E., Sestili F., Savatin D. V. (2021). Biotechnological resources to increase disease-resistance by improving plant immunity: a sustainable approach to save cereal crop production. *Plants*.

[4] Shahzad A., Ullah S., Dar A. A. (2021). Nexus on climate change: agriculture and possible solution to cope future climate change stresses. *Environmental Science and Pollution Research*.

[5] Ali Q., Yu C., Hussain A. (2022). Genome engineering technology for durable disease resistance: recent progress and future outlooks for sustainable agriculture. *Frontiers in Plant Science*.

[6] Mushtaq M., Sakina A., Wani S. H. (2019). Harnessing genome editing techniques to engineer disease resistance in plants. *Frontiers in Plant Science*.

[7] Raghurami Reddy M., Acaso J. T., Alakonya A. E. (2022). Accelerating cereal breeding for disease resistance through genome editing. *Genome editing technologies for crop improvement*.

[8] Turnbull C., Lillemo M., Hvoslef-Eide T. A. (2021). Global regulation of genetically modified crops amid the gene edited crop boom–a review. *Frontiers in Plant Science*.

[9] Bailey-Serres J., Parker J. E., Ainsworth E. A., Oldroyd G. E., Schroeder J. I. (2019). Genetic strategies for improving crop yields. *Nature*.

[10] Tian Z., Wang J. W., Li J., Han B. (2021). Designing future crops: challenges and strategies for sustainable agriculture. *The Plant Journal*.

[11] Grote U., Fasse A., Nguyen T. T., Erenstein O. (2021). Food security and the dynamics of wheat and maize value chains in Africa and Asia. *Frontiers in Sustainable Food Systems*.

[12] Erenstein O., Jaleta M., Mottaleb K. A., Sonder K., Donovan J., Braun H.-J. (2022). Global trends in wheat production, consumption and trade. *Wheat improvement: Food security in a changing climate*.

[13] Langridge P., Stein N., Muehlbauer G. (2018). Economic and Academic Importance of Barley. *The barley genome*.

[14] Guo J., Mao K., Yuan Z. (2021). Global food security assessment during 1961–2019. *Sustainability*.

[15] He G., Zhao Y., Wang L., Jiang S., Zhu Y. (2019). China's food security challenge: effects of food habit changes on requirements for arable land and water. *Journal of Cleaner Production*.

[16] Joshi B. K., Shrestha H. K., Ayer D. K., Ghosh S., Kumari Panda A., Jung C., Singh Bisht S. (2023). Plant Breeding Strategies and Methods for Food Security: Review on the Technology. *Emerging Solutions in Sustainable Food and Nutrition Security*.

[17] Kumar A., Mir R. R., Sehgal D., Agarwal P., Carter A. (2021). Editorial: Genetics and genomics to enhance crop production, towards food security. *Frontiers in Genetics*.

[18] Jeyasri R., Muthuramalingam P., Satish L. (2021). An overview of abiotic stress in cereal crops: negative impacts, regulation, biotechnology and integrated omics. *Plants*.

[19] Kumar S. (2020). Abiotic stresses and their effects on plant growth, yield and nutritional quality of agricultural produce. *International Journal of Food Science and Agriculture*.

[20] Gupta A., Bano A., Pandey N., Rai S., Sharma S., Pathak N. (2021). Recent Advances in Biotic and Abiotic Stresses of *Solanum Melongena* L. *Solanum melongena: Production, Cultivation and Nutrition*.

[21] Fones H. N., Bebber D. P., Chaloner T. M., Kay W. T., Steinberg G., Gurr S. J. (2020). Threats to global food security from emerging fungal and oomycete crop pathogens. *Nature Food*.

[22] Little N. G., DiTommaso A., Westbrook A. S., Ketterings Q. M., Mohler C. L. (2021). Effects of fertility amendments on weed growth and weed–crop competition: a review. *Weed Science*.

[23] Stone P. (2023). The effects of heat stress on cereal yield and quality. *Crop responses and adaptations to temperature stress*.

[24] Stevens G., Motavalli P., Scharf P., Nathan M., Dunn D. (2018). *Crop nutrient deficiencies & toxicities*.

[25] Elouafi I., Shahid M. A., Begmuratov A., Hirich A., Hirich A., Choukr-Allah R., Ragab R. (2020). The Contribution of Alternative Crops to Food Security in Marginal Environments. *Emerging research in alternative crops*.

[26] Trono D., Pecchioni N. (2022). Candidate genes associated with abiotic stress response in plants as tools to engineer tolerance to drought, salinity and extreme temperatures in wheat: an overview. *Plants*.

[27] Munaweera T., Jayawardana N., Rajaratnam R., Dissanayake N. (2022). Modern plant biotechnology as a strategy in addressing climate change and attaining food security. *Agriculture & Food Security*.

[28] Qaim M. (2020). Role of new plant breeding technologies for food security and sustainable agricultural development. *Applied Economic Perspectives and Policy*.

[29] Mohankumar S., Ramasubramanian T. (2014). Role of genetically modified insect-resistant crops in IPM: agricultural, ecological and evolutionary implications. *Integrated pest management*.

[30] Kumar K., Gambhir G., Dass A. (2020). Genetically modified crops: current status and future prospects. *Planta*.

[31] Hafeez U., Ali M., Hassan S. M., Akram M. A., Zafar A. (2023). Advances in breeding and engineering climate-resilient crops: a comprehensive review. *International Journal of Research and Advances in Agricultural Sciences*.

[32] Song X., Yan J., Zhang Y. (2021). Gene flow risks from transgenic herbicide-tolerant crops to their wild relatives can be mitigated by utilizing alien chromosomes. *Frontiers in Plant Science*.

[33] Tyczewska A., Woźniak E., Gracz J., Kuczyński J., Twardowski T. (2018). Towards food security: current state and future prospects of agrobiotechnology. *Trends in Biotechnology*.

[34] Noort M. W., Renzetti S., Linderhof V. (2022). Towards sustainable shifts to healthy diets and food security in sub-Saharan Africa with climate-resilient crops in bread-type products: a food system analysis. *Food*.

[35] Bradshaw J. E. (2017). Plant breeding: past, present and future. *Euphytica*.

[36] Begna T. (2022). Speed breeding to accelerate crop improvement. *International Journal of Agricultural Science and Food Technology*.

[37] Harwood J. (2019). Was the Green Revolution intended to maximise food production?. *International Journal of Agricultural Sustainability*.

[38] Li X., Yadav R., Siddique K. H. (2020). Neglected and underutilized crop species: the key to improving dietary diversity and fighting hunger and malnutrition in Asia and the Pacific. *Frontiers in Nutrition*.

[39] Tadele Z. (2017). Raising crop productivity in Africa through intensification. *Agronomy*.

[40] Li C. (2020). Breeding crops by design for future agriculture. *Journal of Zhejiang University. Science. B*.

[41] Nelson R., Wiesner-Hanks T., Wisser R., Balint-Kurti P. (2018). Navigating complexity to breed disease-resistant crops. *Nature Reviews Genetics*.

[42] Li W., Deng Y., Ning Y., He Z., Wang G.-L. (2020). Exploiting broad-spectrum disease resistance in crops: from molecular dissection to breeding. *Annual Review of Plant Biology*.

[43] Glenn K. C., Alsop B., Bell E. (2017). Bringing new plant varieties to market: plant breeding and selection practices advance beneficial characteristics while minimizing unintended changes. *Crop Science*.

[44] Islam R., Parvin A., Billah M. (2020). Assessment of the effects of genetically modified (GM) foods: a brief study on health and environmental concerns. *Journal of Materials and Environmental Science*.

[45] Ahmar S., Gill R. A., Jung K.-H. (2020). Conventional and molecular techniques from simple breeding to speed breeding in crop plants: recent advances and future outlook. *International Journal of Molecular Sciences*.

[46] Anders S., Cowling W., Pareek A., Gupta K. J., Singla-Pareek S. L., Foyer C. H. (2021). Gaining acceptance of novel plant breeding technologies. *Trends in Plant Science*.

[47] Nicholl D. S. (2023). *An introduction to genetic engineering*.

[48] Khan A., Korban S. S. (2022). Breeding and genetics of disease resistance in temperate fruit trees: challenges and new opportunities. *Theoretical and Applied Genetics*.

[49] Naveed M., Javed J., Waheed U. (2022). The role of CRISPR/Cas9 for genetic advancement of soybean: a review. *Asian Journal of Biotechnology and Genetic Engineering*.

[50] Van Esse H. P., Reuber T. L., van der Does D. (2020). Genetic modification to improve disease resistance in crops. *New Phytologist*.

[51] Vrbničanin S., Božić D., Pavlović D. (2017). Gene flow from herbicide-resistant crops to wild relatives. *Herbicide resistance in crops and weeds*.

[52] Álvarez-Alfageme F., Devos Y., Camargo A. M., Arpaia S., Messéan A. (2022). Managing resistance evolution to transgenic Bt maize in corn borers in Spain. *Critical Reviews in Biotechnology*.

[53] Green J. M. (2018). The rise and future of glyphosate and glyphosate-resistant crops. *Pest Management Science*.

[54] Malenica N., Dunić J. A., Vukadinović L., Cesar V., Šimić D. (2021). Genetic approaches to enhance multiple stress tolerance in maize. *Genes*.

[55] Gupta S., Kumar A., Patel R., Kumar V. (2021). Genetically modified crop regulations: scope and opportunity using the CRISPR-Cas9 genome editing approach. *Molecular Biology Reports*.

[56] Dwiningsih Y., Al-Kahtani J. (2022). Genome-wide association study of complex traits in maize detects genomic regions and genes for increasing grain yield and grain quality. *Advance Sustainable Science Engineering and Technology*.

[57] Singh R. K., Prasad A., Muthamilarasan M., Parida S. K., Prasad M. (2020). Breeding and biotechnological interventions for trait improvement: status and prospects. *Planta*.

[58] Zafar M. M., Razzaq A., Farooq M. A. (2020). Insect resistance management in Bacillus thuringiensis cotton by MGPS (multiple genes pyramiding and silencing). *Journal of Cotton Research*.

[59] Ali Q., Malik A. (2021). Genetic response of growth phases for abiotic environmental stress tolerance in cereal crop plants. *Genetika*.

[60] Lata C., Shivhare R. (2021). Engineering cereal crops for enhanced abiotic stress tolerance. *Proceedings of the Indian National Science Academy*.

[61] Wang G., Su H., Abou-Elwafa S. F. (2023). Functional analysis of a late embryogenesis abundant protein ZmNHL1 in maize under drought stress. *Journal of Plant Physiology*.

[62] Wijewardene I., Shen G., Zhang H. (2021). Enhancing crop yield by using Rubisco activase to improve photosynthesis under elevated temperatures. *Stress Biology*.

[63] Dimaano N. G., Iwakami S. (2021). Cytochrome P450-mediated herbicide metabolism in plants: current understanding and prospects. *Pest Management Science*.

[64] Bowerman A. F., Byrt C. S., Roy S. J. (2023). Potential abiotic stress targets for modern genetic manipulation. *The Plant Cell*.

[65] Ghimire B. K., Yu C. Y., Kim W.-R. (2023). Assessment of benefits and risk of genetically modified plants and products: current controversies and perspective. *Sustainability*.

[66] Uddin T. M., Chakraborty A. J., Khusro A. (2021). Antibiotic resistance in microbes: history, mechanisms, therapeutic strategies and future prospects. *Journal of Infection and Public Health*.

[67] Begna T. (2021). Conventional breeding methods widely used to improve self-pollinated crops. *International Journal of Research*.

[68] Campos E. V., Proença P. L., Oliveira J. L., Bakshi M., Abhilash P., Fraceto L. F. (2019). Use of botanical insecticides for sustainable agriculture: future perspectives. *Ecological Indicators*.

[69] Harfouche A. L., Petousi V., Meilan R., Sweet J., Twardowski T., Altman A. (2021). Promoting ethically responsible use of agricultural biotechnology. *Trends in Plant Science*.

[70] Tepliuk M., Sahaidak M., Petrishyna T., Fokina-Mezentsev K., Fomenko B., Vasyliev I. (2023). Managing of responsible consumption and sustainable production enterprises in the glocalization conditions. *Acta Innovations*.

[71] Gouveia B. C., Calil I. P., Machado J. P. B., Santos A. A., Fontes E. P. (2017). Immune receptors and co-receptors in antiviral innate immunity in plants. *Frontiers in Microbiology*.

[72] Shittu H. O., Aisagbonhi E., Onyemaechi H. O. (2019). PLANTS’ INNATE defence mechanisms against phytopathogens. *The Journal of Microbiology, Biotechnology and Food Sciences*.

[73] Noman A., Aqeel M., Lou Y. (2019). PRRs and NB-LRRs: from signal perception to activation of plant innate immunity. *International Journal of Molecular Sciences*.

[74] Sun L., Zhang J. (2020). Regulatory role of receptor-like cytoplasmic kinases in early immune signaling events in plants. *FEMS Microbiology Reviews*.

[75] Yang C., Dolatabadian A., Fernando W. D. (2022). The wonderful world of intrinsic and intricate immunity responses in plants against pathogens. *Canadian Journal of Plant Pathology*.

[76] Vinod K., Sabah A. (2018). Plant defense against pathogens: the role of salicylic acid. *Research Journal of Biotechnology*.

[77] Bartsch L. M., Damasio M. P., Subudhi S., Drescher H. K. (2020). Tissue-resident memory T cells in the liver—unique characteristics of local specialists. *Cells*.

[78] Hou S., Liu Z., Shen H., Wu D. (2019). Damage-associated molecular pattern-triggered immunity in plants. *Frontiers in Plant Science*.

[79] Poltronieri P., Brutus A., Reca I. B., Francocci F., Cheng X., Stigliano E. (2020). Engineering plant leucine rich repeat-receptors for enhanced pattern-triggered immunity (PTI) and effector-triggered immunity (ETI). *Applied plant biotechnology for improving resistance to biotic stress*.

[80] Zarrabi A., Perrin D., Kavoosi M. (2023). Rhabdomyosarcoma: current therapy, challenges, and future approaches to treatment strategies. *Cancers*.

[81] Silva R. N., Monteiro V. N., Steindorff A. S., Gomes E. V., Noronha E. F., Ulhoa C. J. (2019). Trichoderma/pathogen/plant interaction in pre-harvest food security. *Fungal Biology*.

[82] Smith S. (2019). The foundations, continuing evolution, and outcomes from the application of intellectual property protection in plant breeding and agriculture. *Plant Breeding Reviews*.

[83] Clancy M. S., Moschini G. (2017). Intellectual property rights and the ascent of proprietary innovation in agriculture. *Annual Review of Resource Economics*.

[84] Kock M. A. (2021). Open intellectual property models for plant innovations in the context of new breeding technologies. *Agronomy*.

[85] Jenney P. B. (2022). *Keeping what you sow: Intellectual property rights for plant breeders and seed growers*.

[86] Kock M. A. (2022). Plant breeding and intellectual property: a controversial topic. *Intellectual property protection for plant related innovation: Fit for Future?*.

[87] Smulders M. J., van de Wiel C. C., Lotz L. A. (2021). The use of intellectual property systems in plant breeding for ensuring deployment of good agricultural practices. *Agronomy*.

[88] Jacquet F., Jeuffroy M.-H., Jouan J. (2022). Pesticide-free agriculture as a new paradigm for research. *Agronomy for Sustainable Development*.

[89] Cholchawalit K., Jaiharn N. (2017). *Thai legal control and liability of living modified organisms: a case study of LMO products*.

[90] Pereira R. (2022). The Cartagena protocol on biosafety and the regulation of transboundary movement of living modified organisms. *Transgenic insects: Techniques and applications*.

[91] Akinbo O., Obukosia S., Ouedraogo J. (2021). Commercial release of genetically modified crops in Africa: interface between biosafety regulatory systems and varietal release systems. *Frontiers in Plant Science*.

[92] Beeckman D. S., Rüdelsheim P. (2020). Biosafety and biosecurity in containment: a regulatory overview. *Frontiers in Bioengineering and Biotechnology*.

[93] Sebesta J., Xiong W., Guarnieri M. T., Yu J. (2022). Biocontainment of genetically engineered algae. *Frontiers in Plant Science*.

[94] EFSA Panel on Genetically Modified Organisms (GMO) (2011). Guidance on the post-market environmental monitoring (PMEM) of genetically modified plants. *EFSA Journal*.

[95] Kafi M. J. (2022). *Regulation of Genetically Modified Organisms in India and the Influence of Cartagena Protocol on its Biosafety Framework*.

[96] Brown C. A., Dunn J. J. (2024). Laboratory safety. *Clinical laboratory management*.

[97] Gao G. F. (2019). For a better world: biosafety strategies to protect global health. *Biosafety and Health*.

[98] Kallergi A., Asin-Garcia E., Martins dos Santos V. A., Landeweerd L. (2021). Context matters: on the road to responsible biosafety technologies in synthetic biology. *EMBO Reports*.

[99] Akram F., Sahreen S., Aamir F. (2023). An insight into modern targeted genome-editing technologies with a special focus on CRISPR/Cas9 and its applications. *Molecular Biotechnology*.

[100] Sudheer S., Bai R. G., Usmani Z., Sharma M. (2020). Insights on engineered microbes in sustainable agriculture: biotechnological developments and future prospects. *Current Genomics*.

[101] Raza A., Razzaq A., Mehmood S. S. (2019). Impact of climate change on crops adaptation and strategies to tackle its outcome: a review. *Plants*.

